# Protocol for developing a melanoma brain metastasis mouse model for preclinical drug testing and brain tumor progression monitoring

**DOI:** 10.1016/j.xpro.2025.104081

**Published:** 2025-09-11

**Authors:** Jared Almazan, Tursun Turapov, Sheri L. Holmen

**Affiliations:** 1 Huntsman Cancer Institute, University of Utah Health Sciences Center, Salt Lake City, UT 84112, USA; 2 Department of Oncological Sciences, University of Utah Health Sciences Center, Salt Lake City, UT 84112, USA; 3 Department of Surgery, University of Utah Health Sciences Center, Salt Lake City, UT 84112, USA

**Keywords:** Cancer, Health Sciences, Model Organisms, Molecular Biology

## Abstract

We present a protocol to generate a murine model of established melanoma brain metastases by intracranially injecting tumor cells into newborn “glowing head” C57BL/6 syngeneic mice, which are tolerized to both GFP and luciferase. First, we describe steps for cell and animal preparation. We then detail the utilization of bioluminescent imaging to monitor brain tumor progression. This model enables the evaluation of therapeutic efficacy against established melanoma brain metastases.

For complete details on the use and execution of this protocol, please refer to Almazan et al.^1^

## Highlights

• Develop a murine model of melanoma brain metastases for preclinical drug testing
• Intracranial injections into newborn mice to induce melanoma brain metastases
• Detection of brain tumor growth and progression using bioluminescent imaging

## Abstract (editor highlights)

Publisher’s note: Undertaking any experimental protocol requires adherence to local institutional guidelines for laboratory safety and ethics.

## Graphical abstract

**Figure:**
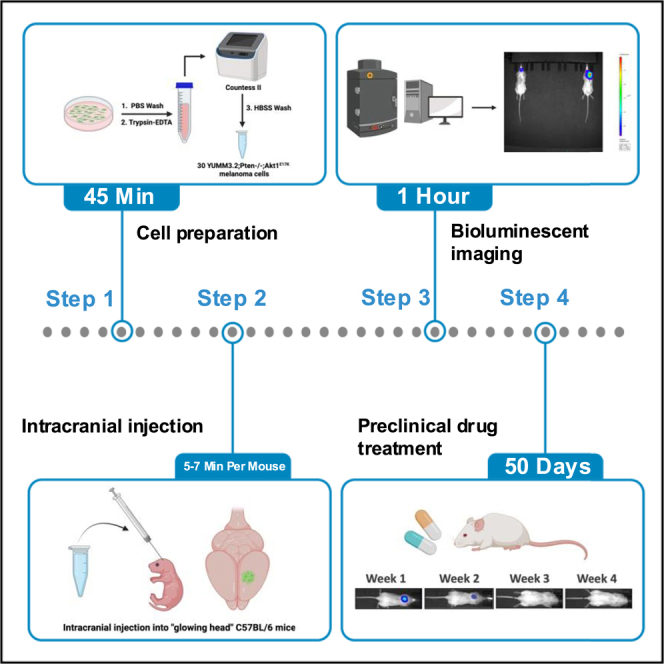

## Before you begin

Melanoma is one of the deadliest forms of skin cancer, with brain metastases being the major cause of treatment failure.^2^ To address this issue and evaluate the effects of drug treatments on established brain metastases, this protocol outlines the steps for generating a murine model of melanoma brain metastases using GFP and luciferase-expressing syngeneic YUMM3.2;Pten−/−;Akt1^E17K^ melanoma cells in newborn “glowing head” C57BL/6 mice.^1^

The expression of GFP in this modified cell line has the advantage of confirming delivery of the virus harboring AKT1^E17K^ and luciferase, with the latter being essential for the bioluminescent imaging (BLI) technique. Additionally, this cell line expresses BRAF^V600E^, which activates the MAPK pathway, and expresses AKT1^E17K^ in the context of *Cdkn2a* and *Pten* loss, resulting in activation of the PI3K/AKT signaling pathway, which is a common occurrence in human melanoma. Furthermore, we have previously demonstrated that PI3K/AKT pathway activation in this context is required for melanoma cell growth in the brain.^3^

This protocol also takes advantage of the immunocompetent “glowing head” C57BL/6 mice engineered by Day et al., as this mouse strain is tolerized to both GFP and luciferase.^4^ Additionally, the albino phenotype of these mice reduces the effects of autofluorescence. For this model, we intracranially inject 30 YUMM3.2;Pten−/−;Akt1^E17K^ melanoma cells into newborn mice, as previous pilot studies with injections of 1000 and 100 cells resulted in early mortality, thus prompting the use of a smaller number of cells. Furthermore, newborn mice are utilized because their skull is softer and injections can be done with a gas tight Hamilton syringe without the need for a stereotactic injection device. Lastly, bioluminescent imaging will be used to detect the establishment and colonization of the cell line in the brain, as well as monitor tumor burden over time.

Various models have been developed to study melanoma, including zebrafish models for melanoma development and drug screening, and patient-derived xenografts for assessing drug resistance and treatment efficacy.^5^ However, there is a lack of preclinical models that replicate the brain metastatic patterns seen in patients. Our model utilizes an engineered melanoma cell line that closely mimics human brain metastases and enables direct induction of tumors into the brains of immunocompetent mice. Therefore, the generation of this model facilitates efficient modeling of melanoma brain metastases for preclinical drug studies.

### Institutional permissions

All experiments performed on animal models were approved by the institutional animal care and use committee (IACUC) at the University of Utah. Please obtain all permissions needed for the use of animal models from proper authorities before performing any experiments or procedures on live animals.

### Cell culture preparation

**Timing: 1–3 days**

**Figure 1:**
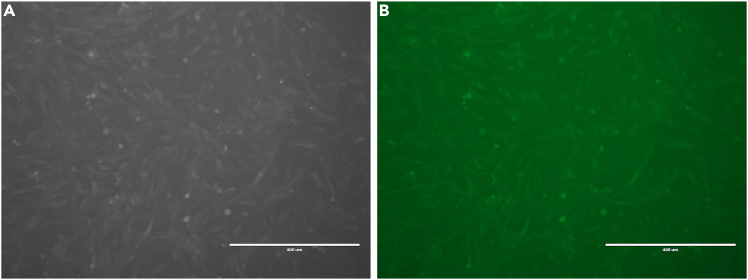
GFP expression in the YUMM3.2;Pten−/−;Akt1^E17K^ melanoma cells (A and B) Using a fluorescence microscope, GFP expression is confirmed in the cell line (A) with the GFP filter (B).

***Note:*** We recommend achieving approximately 80% cell confluency prior to cell injection, as over confluency can compromise the viability of the cells.

1. Thaw the GFP and luciferase-expressing YUMM3.2;Pten−/−;Akt1^E17K^ melanoma cells and culture them in Dulbecco’s Modified Eagle Medium (DMEM)/F-12 supplemented with 10% FBS, 1% Penicillin-Streptomycin, and 1% Minimum Essential Medium (MEM) Non-Essential Amino Acids Solution in a 10 cm tissue culture dish.
2. Maintain cells in culture at 37°C with 5% CO_2_ for 1–3 days.
3. Prepare for the injection when the preferred confluency is achieved.
4. Using a fluorescence microscope, confirm the presence of GFP expression in the cells as shown in Figure 1. Successful GFP expression also confirms the presence of luciferase in the cell line, which is crucial in the upcoming steps of the protocol. Furthermore, a luciferase assay may be performed to confirm luciferase activity in the cell line.

## Key resources table

**Table:** 

REAGENT or RESOURCE	SOURCE	IDENTIFIER
**Chemicals, peptides, and recombinant proteins**		

DMEM/F-12, HEPES	Thermo Fisher Scientific	11330057
Trypsin-EDTA (0.05%), phenol red	Thermo Fisher Scientific	25300120
Fetal bovine serum	Atlas Biologicals Inc.	F-0500-DR
Penicillin-streptomycin (10,000 U/mL)	Thermo Fisher Scientific	15140122
MEM non-essential amino acids solution (100X)	Thermo Fisher Scientific	11140050
Isoflurane inhalation solution 99.9%	McKesson Medical-Surgical, Inc.	803250
PBS, pH 7.4	Thermo Fisher Scientific	10010023
D-luciferin, potassium salt	Gold Biotechnology Inc.	LUCK-2G
HBSS, calcium, magnesium, no phenol red	Thermo Fisher Scientific	14025134
Trypan blue stain	Thermo Fisher Scientific	T10282

**Experimental models: Cell lines**		

YUMM3.2 (PTEN^−/−^, Akt1^E17K^, luciferase, EGFP)	Almazan et al.^1^	N/A

**Experimental models: Organisms/strains**		

C57BL/6-*Tyr*^*c-Brd*^ Tg(Gh1-luc/EGFP) D8Mrln/J; C57BL/6 glowing head	Day et al.^4^; The Jackson Laboratory	Strain #027662

**Software and algorithms**		

Living Image Software	Revvity	https://www.revvity.com/software-downloads/in-vivo-imaging

**Other**		

Thermo Fisher Countess II automated cell counter	Marshall Scientific	I-CACC2
Countess cell counting chamber slide	Thermo Fisher Scientific	C10228
IVIS spectrum *in vivo* imaging system	Revvity	CLS158738
Gas-tight Hamilton syringe	Hamilton Company	80075
EVOS FL fluorescence microscope	Thermo Fisher Scientific	AMF4300
Olympus CKX53-BSC tissue culture inverted microscope	Evident Scientific	CKX53-BSC
Easy Touch U-100 insulin syringe with needle, 25G 1 cc 1/2 in (12.7 mm)	Amazon	N/A

## Step-by-step method details

### Preparation of cells for intracranial injection

**Timing: 45 min**

This step prepares tumor cells for injection with the desired cell density and volume.

1. Remove the media from the 10 cm tissue culture dish of ∼80% confluent cells with gentle aspiration.
  a. Wash cells once in 1 × PBS.
  b. Add 1 mL of 0.05% Trypsin-EDTA and swirl the tissue culture dish to completely cover the cells.
  c. Incubate the tissue culture dish at 37°C for 5–7 min.
  d. Check under the microscope to see if the cells have detached from the tissue culture dish.

***Note:*** It is critical to ensure that all the cells have detached from the plate before proceeding to the next step.

2. Add 5 mL of media to stop the Trypsin-EDTA reaction.
  a. Rinse the media over the tissue culture dish several times to ensure that all the cells have been collected from the plate.
  b. Transfer the cells into a sterile 15 mL conical tube.
  c. Centrifuge the cells for 4 min at 400 × *g* at room temperature (RT).
  d. Remove the supernatant with gentle aspiration.
  e. Gently resuspend the cell pellet in 1 mL of fresh media.
3. Determine the cell concentration using a Countess II Automated Cell Counter per the manufacturer’s specifications. In this step, the cell viability and cell concentration are measured.
  a. In a sterile microcentrifuge tube, add 10 μL of 0.4% Trypan Blue stain.
  b. Resuspend and transfer 10 μL of the cell suspension from Step 2 into the same tube with the Trypan Blue.
  c. Resuspend to mix the Trypan Blue with the cell suspension.
  d. Transfer 10 μL from the mixture into a chamber of a countess cell counting chamber slide.
  e. Insert the slide into the Countess II Automated Cell Counter and quantify the concentration of live cells in the cell suspension.
  f. Record the live cell concentration.

***Note:*** Manual counting with a hemocytometer is an alternative method to determine the cell concentration if an automated counter is not available.

4. Determine the volume of cells required to inject 30 cells into each newborn mouse.
  ***Note:*** The calculations in this preparation step are designed to resuspend the cells in a final volume of 10 mL (demonstrated in steps 4 and 5), as using a larger volume ensures the accurate delivery of the intended number of cells to reliably induce brain metastases.
  a. Calculate the volume of cell suspension needed to inject 30 cells per mouse.
    ***Note:*** For example, live cell concentration = 8.67 × 10^6^ cells/mL.
    i. 10 mL of cell suspension = 10,000 μL.
         10 cells per μL density.
         Each mouse is injected with 30 cells (3 μL = 30 cells).
    ii. Perform calculations to obtain 100,000 cells:
         10,000 μL × $\frac{10\;cells}{1\;\mu L}$ = 100,000 cells.
         100,000 cells × $\frac{1\;mL}{8.64\;x\;10^{6}\;cells}$ = 0.01157 mL = **11.57 μL.**
         **11.57 μL** = 100,000 cells (this volume will be transferred into a final volume of 10 mL in the next step).
         3 μL = 30 cells.
5. Wash and prepare the cells for injection.
  a. Transfer the volume calculated from the previous step into a 1.5 mL microcentrifuge tube.
  b. Add 100 μL of 1× HBSS into the tube and resuspend.
  c. Transfer the resuspended cells into a 15 mL conical tube with ∼5–10 mL of 1X HBSS and spin down the tube for 2 min at 450 g at RT.
  d. Gently aspirate the supernatant and resuspend the pellet in 100 μL of 1× HBSS.
  e. Add ∼5–10 mL of 1X HBSS and repeat the spin down as previously described.
  f. Gently aspirate the supernatant and resuspend the pellet in 100 μL of 1× HBSS but then transfer the cell suspension into a 15 mL conical tube with 9900 μL of 1X HBSS. Vortex and invert afterward.
  g. Transfer 5 mL of the cell suspension into a clean 5 mL Eppendorf tube and immediately place the tube on ice.
  h. Prepare two 1.5 mL microcentrifuge tubes for the following step. One tube with 1 mL of 1X HBSS, and the other with 1 mL of 70% EtOH.

### Intracranial injection into newborn “glowing head” C57BL/6 mice

**Timing: 5–7 min per mouse**

This step describes the injection process into newborn “glowing head” C57BL/6 mice. Newborn mice can be injected ranging from postnatal Day 0–Day 3.

**Figure 2:**
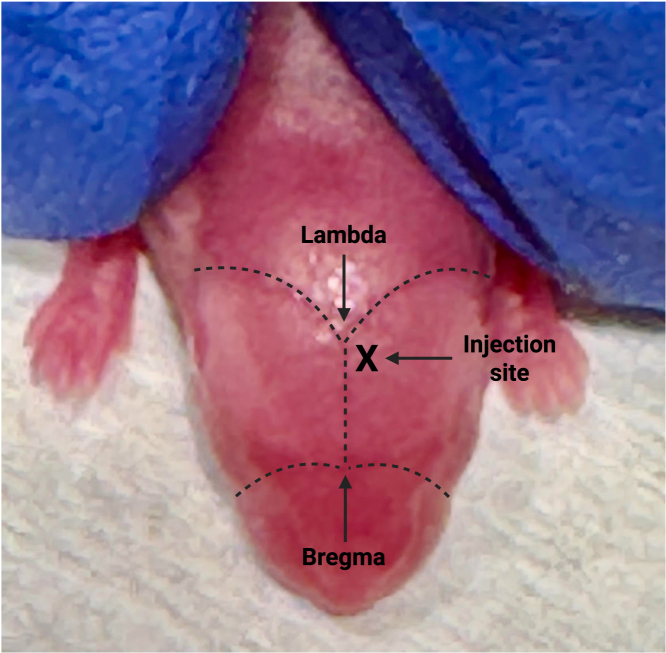
Dorsal view of a newborn mouse This figure shows the location of the injection site in reference to lambda and bregma.
    **Figure 3:**
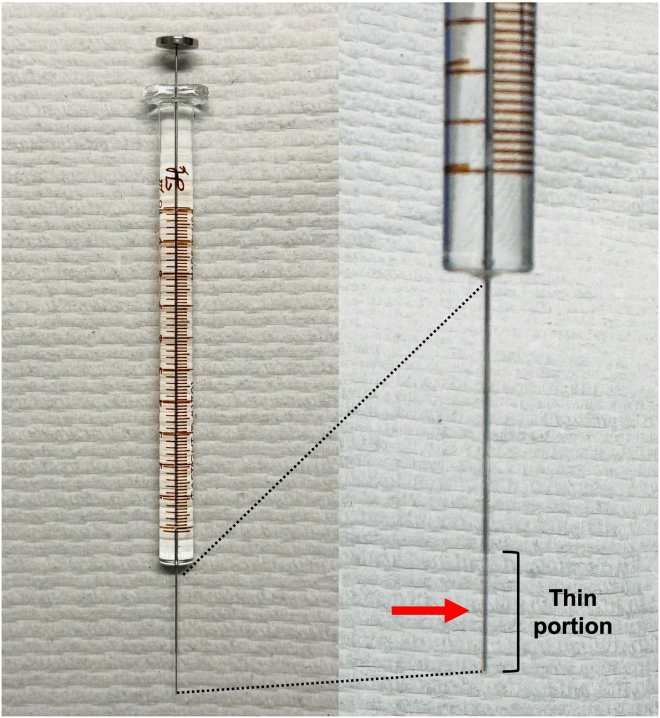
Image of a gas-tight Hamilton syringe used for intracranial injections The thin portion of the syringe is indicated along with the depth of the injection (red arrow).
  d. Slowly inject the cells into the skull by pressing down on the plunger. Hold the needle in place for ∼3 seconds and then slowly retract the needle.
  e. Between each injection, repeat the wash steps for the Hamilton syringe as previously described in Step 6.

6. Clean the gas-tight Hamilton syringe prior to each injection.
  a. Using the Hamilton syringe, draw up 70% EtOH from the 1.5 mL microcentrifuge tube and eject it back into the tube. Repeat this multiple times.
  b. Repeat the previous step but with the 1.5 mL microcentrifuge tube containing 1 × HBSS.
7. Inject the newborn pups with the GFP and luciferase-expressing YUMM3.2;Pten−/−;Akt1^E17K^ melanoma cells.
  a. While wearing gloves, place a drop of the 70% EtOH on the top of the head of the newborn pup to sterilize the area.
  b. With the empty Hamilton syringe, slowly draw up 3 μL (30 cells) from the cell suspension in Step 5.
  c. On a flat surface, gently hold the pup around the shoulder area and insert the syringe into the skull 2-mm ventral and anterior to lambda (intersection of the lambdoid and sagittal sutures) as shown in Figure 2.
    ***Note:*** Insert the syringe into the injection site at a depth of halfway through the thin portion (Figure 3).

### Assess brain tumor formation with bioluminescent imaging

**Timing: 15 min per cage**

This step confirms and measures brain tumor growth via BLI. Once a signal is detected, the mice can be enrolled into preclinical drug studies to evaluate the efficacy of various drug treatments in suppressing established melanoma brain metastases.

Duration: Weekly until the experimental endpoint is reached.

8. Gather all the necessary materials including insulin syringes, D-Luciferin (16.7 mg/mL), bucket of ice, flash drive, and paper towels.
9. Thaw D-Luciferin on ice for 10-20 min.

***Note:*** Once thawed, keep the D-luciferin on ice and protect it from light. This can be done by covering the ice bucket, using light-protected microcentrifuge tubes, or wrapping the tube in tinfoil.

**Figure 4:**
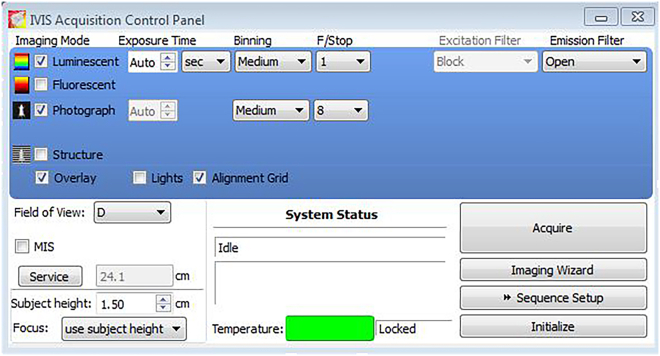
Image of the control panel for the Living Image software
11. The mice are first imaged upon weaning (day 21).
12. Place the mice in the induction chamber with 2.5% isoflurane and 2 L/min oxygen for ∼5 min for the mice to be anesthetized.
13. After the mice have been anesthetized, inject 100 μL of D-Luciferin intraperitoneally into each mouse.

***Note:*** If bubbles appear in the syringe from drawing up the D-Luciferin, tap the syringe and slowly dispense the solution to eliminate the air. Repeat until no bubbles are present in the syringe.

***Note:*** After the intraperitoneal injections, transfer the mice to a pre-heated animal imaging platform inside the IVIS chamber and maintain anesthesia via the nose cone.

10. Turn on and set up the IVIS Spectrum In Vivo Imaging System per the manufacturer’s specifications.
  a. Open the Living Image software.
  b. Log into the user account.
  c. Turn on the oxygen concentrator and isoflurane vaporizer to initiate the flow of oxygen and anesthetic into the induction chamber. Prepare the nose cones inside the pre-heated animal imaging platform in the IVIS chamber.
    i. Adjust the flow rate of isoflurane to be at 2.5 v/v.
    ii. Adjust the oxygen flow rate to 2 L/min.
  d. Click on “Initialize” to warm-up the IVIS system.
    i. Initializing is finished once the temperature bar turns from red to green.
  e. Set up the Field of View.
    i. For whole-body BLI, choose “D”. For *ex vivo* organ BLI, choose “C”.
  f. Set exposure time to “Auto” and keep the Subject Height at 1.50 cm.
  g. Check the Luminescent and Photograph boxes in Imaging Mode.
  h. Final set up of the control panel is shown in Figure 4.

14. Acquire bioluminescent images at 10 min post-injection.
  a. Click on “Acquire” in the control panel to image the mice.

***Note:*** Space the mice at least one nose cone distance away from one another to prevent interfering bioluminescence. An alternative is to use dividers between each mouse.

**Figure 5:**
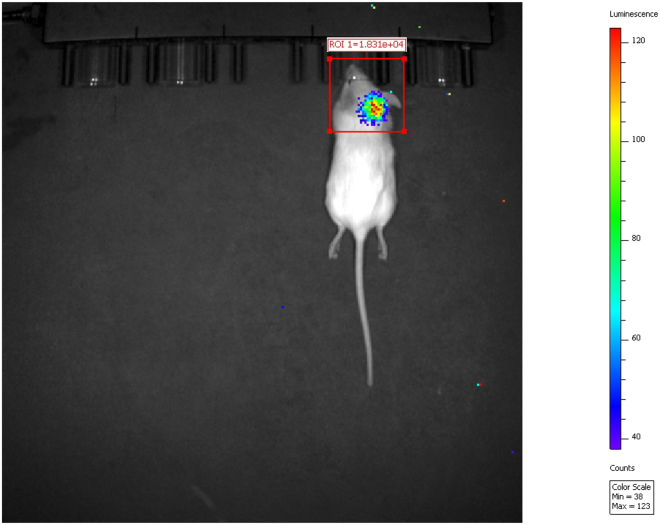
Representative BLI of 5-week-old “glowing head” C57BL/6 mouse with a brain tumor undergoing targeted therapy An ROI box is placed around the head of the mouse and the BLI signal is quantified with radiance. Upon quantification, the BLI signal of the mouse in this figure is 18,310 radiance (p/sec/cm^2^/sr).

***Note:*** Radiance is preferred over total flux, as it normalizes both the surface area and bioluminescent signal intensity, thereby enabling more accurate longitudinal comparisons of data from the same animal.

15. Quantify the bioluminescence signal of each mouse.
  a. In the captured image, set the count to Radiance.
  b. In the Tool Palette pop-up, select “ROI Tools”, and place an ROI (region of interest) box around the head area each mouse (site of which the tumor will develop) and click on “Acquire”. This quantifies the signal with radiance (p/sec/cm^2^/sr) (Figure 5).

16. After image acquisition, transfer the mice back to their cage and monitor for recovery from anesthesia.

***Note:*** The mice will awaken and mobilize within a few minutes.

17. The mice can be imaged weekly (timing varies for what is appropriate for the proposed study) for a predetermined number of days to evaluate the effects of the drug treatment on established melanoma brain metastases.

***Note:*** Once a BLI signal is detected (∼21 days of age), the mice can be enrolled into preclinical drug studies and may receive treatment via oral gavage, intravenous (IV) injection, and/or intraperitoneal injections.

## Expected outcomes

This protocol describes the development of a murine model of established melanoma brain metastases. Following the completion of this protocol, the YUMM3.2;Pten−/−;Akt1^E17K^ melanoma cells will successfully grow in the brain, and 100% of the mice will develop a BLI signal in the brain (Figure 5). It is anticipated that the mice in this model will develop a BLI signal by weaning (day 21); however, in rare cases, some mice may require additional days post-weaning to exhibit a BLI signal. Subsequent imaging after approximately three days can provide more time for tumor cell detection.

**Figure 6:**
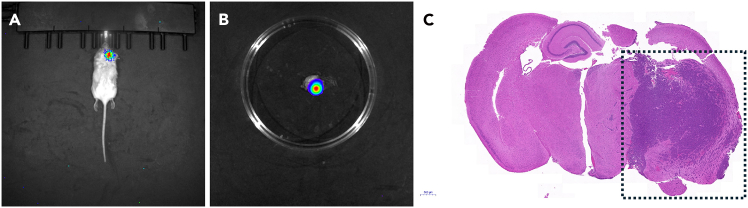
Representative images of the growth of the YUMM3.2;Pten−/−;Akt1^E17K^ melanoma cells in the brain (A–C) Images of a whole-body BLI (A), ex vivo BLI following euthanasia that confirms the successful growth of the cells in the brain (B), and an H&E stain on the brain tissue with the tumor cells indicated by the dashed box (C).

Additionally, the BLI signal correlates with brain tumor growth as assessed histologically after necropsy, as shown in Figure 6. Once a BLI signal is detected, it is crucial to initiate the proposed preclinical drug study immediately, as the mice begin to decline rapidly with brain tumor growth. Signs of declining health may include heavy breathing, squinting eyes, running in circles, ungroomed fur, and lack of mobility.

This model was designed to mimic clinical cases of established melanoma brain metastases and to evaluate the efficacy of treatment combinations in suppressing metastatic brain tumor growth. Compounds that successfully attenuated tumor growth in the brain and prolonged survival in this model include the FAK inhibitor VS-4718 and the RAF/MEK clamp avutometinib in combination.^1^ Other treatment strategies have yet to be evaluated in this model.

## Limitations

A limitation of this model is that it does not fully represent the biology of all melanoma patients with established brain metastases, as this model is driven by mutant BRAF in the context of Akt1^E17K^ along with *Pten* and *Cdkn2a* loss. Consequently, preclinical studies that evaluate treatment strategies against other melanoma subtypes – such as those with mutant NRAS, NF1 loss, or triple wild-type have yet to be tested in this context. Furthermore, BLI in this protocol only measures luminescence exclusively from the melanoma cells injected intracranially and monitors changes longitudinally. We suggest incorporating additional techniques such as magnetic resonating imaging (MRI) alongside BLI and histological analysis of the tissue, if the precise localization of cells, tumor volume, and tumor characteristics are critical to the study. Lastly, this model specifically represents brain metastases originating from cutaneous melanoma and has not been tested on cells from uveal or mucosal melanoma.

## Troubleshooting

### Problem 1

Mice do not survive up to weaning (Day 21).

### Potential solution

If the mice do not survive up to the weaning date, this can be due to several reasons. As previously mentioned, an injection of 30 cells was determined due to the aggressiveness of the cell line; injecting a higher number of cells may lead to early mortality. Injecting 30 cells was sufficient to allow survival up to 4 weeks. This highlights the importance of accurately counting the viable melanoma cells and using calibrated instruments. To ensure accurate cell counts, gently resuspend and/or invert the tube several times before quantification. Avoid inverting too quickly since this may lead to air bubble formation. Additionally, it is possible that the injection was performed too deep. Therefore, it is crucial to inject the syringe only 2 mm ventral to bregma as shown in Figure 3.

### Problem 2

The mice do not produce a BLI signal upon weaning.

### Potential solution

**Figure 7:**
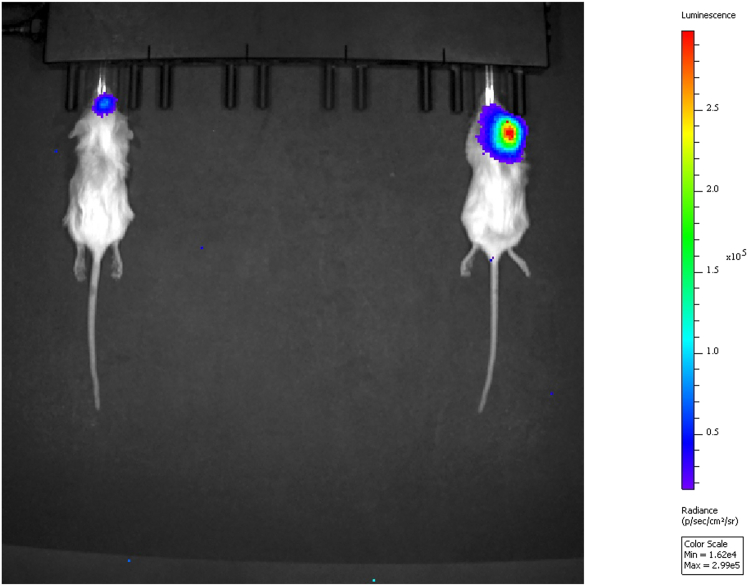
Mouse placement in the IVIS chamber Representative image of proper spacing (at least one nose-cone distance apart) of the mice to prevent interfering bioluminescent signals. An alternative strategy is to use dividers.

If the mice do not produce a signal at weaning, one explanation is that they could be positioned too closely during imaging in the IVIS system. As previously mentioned, spacing the mice at least one nose-cone distance apart prevents interfering bioluminescent signals that might obscure the signal from other mice. An example of proper mouse spacing is shown in Figure 7. If it is necessary to use all of the nose-cone slots in the IVIS chamber, dividers can be added between each mouse to reduce signal interference.

It is also critical to image the mice 10 minutes post-intraperitoneal injection of luciferin, as described in the protocol, due to the time required for the substrate to reach the tumor cells. In rare instances, the mice may require an additional few days to produce a detectable BLI signal. If a mouse does not produce a signal on day 21 (weaning), repeat the BLI after three days to allow more time for the tumor cells to be detected.

## Resource availability

### Lead contact

Further information and requests for resources and reagents should be directed to and will be fulfilled by the lead contact, Dr. Sheri Holmen (sheri.holmen@hci.utah.edu).

### Technical contact

Technical questions on executing this protocol should be directed to and will be answered by the technical contact, Jared Almazan (jared.almazan@hci.utah.edu).

### Materials availability

The YUMM3.2;Pten−/−;Akt1^E17K^ melanoma cell line utilized throughout the protocol was engineered by members of the Holmen lab through modification of isogenic YUMM3.2 cells. For access to this cell line, please contact Jared Almazan or Dr. Sheri Holmen.

### Data and code availability

This study did not generate or analyze datasets or code.

## Acknowledgments

The work was supported by the funds provided by the 10.13039/100010637Huntsman Cancer Foundation and grants from the 10.13039/100000002National Institutes of Health (NIH) (R01CA121118). We thank the Precision Cancer Models (PCM) Shared Resource at Huntsman Cancer Institute at the University of Utah for allowing our lab to utilize the IVIS spectrum *in vivo* imaging system for these studies.

## Author contributions

Writing, review, and/or revision of the manuscript: J.A. Graphical abstract/figure generation: J.A. Development of the methodology: T.T. Utilization and initiation of the model: J.A. and T.T. Funding and manuscript review: S.L.H.

## Declaration of interests

The authors declare no competing interests.
